# Overexpression of CXCR7 is a Novel Indicator for Enzalutamide Resistance in Castration-Resistant Prostate Cancer Patients

**DOI:** 10.1155/2021/6649579

**Published:** 2021-08-06

**Authors:** Yong Luo, Qiankun Li, Xiaobing Yang, Dechao Wei, Bingfu Feng, Mingchuan Li, Yili Han, Jiahui Zhao, Yunhua Lin, Qing Li, Zhu Hou, Hongyu Zhuang, Yongguang Jiang

**Affiliations:** Department of Urology, Beijing Anzhen Hospital, Capital Medical University, Anzhenli Street, Chaoyang District, Beijing 100029, China

## Abstract

**Background:**

To evaluate whether the overexpression of chemokine receptor-7 (CXCR7) in prostatic tissues obtained from men with Castration-Resistant Prostate Cancer (CRPC) is associated with resistance to enzalutamide (Enza).

**Methods:**

Based on the inclusion criteria of CRPC in EAU guidelines, all eligible patients treated in our hospital from January 2015 to December 2019 were included. Cases underwent radical prostatectomy, docetaxel-based chemotherapy, or new endocrine therapies (including Enza or abiraterone), and cases with severe cardiopulmonary disease or other malignant tumors were excluded. After immunohistochemical staining for CXCR7 expression in prostatic biopsy tissues, all enrolled cases were divided into two groups, namely, the CXCR7-positive group and the CXCR7-negative group. And then, PSA response to Enza treatment was recorded in detail and comparatively analyzed. In addition, the Cox proportional hazard modeling and the Kaplan-Meier analysis were used to determine PSA progression-free survival (PSAP-FS) and clinical or radiographic progression-free survival (CRP-FS) in this cohort.

**Results:**

A total of 79 CRPC individuals were enrolled and evaluated in this study. Median follow-up durations were 24 months (range, 12-42) in the CXCR7-positive group (*n* = 47) and 28.5 months (range, 12-42) in the CXCR7-negative group (*n* = 32). The patients with lower CXCR7 expression showed much better PSA response to Enza treatment. There was 84.4% of CXCR7- cases showing decreasing PSA response, while there were 71.4% in the CXCR7/1+ group and 31.2% in the CXCR7/2+ group, respectively. All patients in the CXCR7/3+ group showed increasing PSA response to Enza treatment. And the percentage of patients whose PSA decreased over 50% is significantly higher in the CXCR7-negative group than in the CXCR7-positive group (68.8% vs. 8.5%, *P* < 0.001), and the percentage of patients whose PSA decreased over 90% is also remarkably higher in the CXCR7-negative group (43.8% vs. 0, *P* < 0.001). The Kaplan-Meier analysis demonstrated that the oncologic outcomes of CXCR7-negative patients were improved much significantly by Enza treatment in comparison with those of CXCR7-positive patients. Significantly increased median PSAP-FS (21 months vs. 6 months, *P* < 0.0001) and CRP-FS (27 months vs. 9 months, *P* < 0.0001) were obtained in the CXCR7-negative group. The further stratified analysis in all CXCR7-positive patients demonstrated that the patients with higher CXCR7 expression showed much worse outcome. The median time of PSAP-FS was 21 months in the CXCR7/1+ group, 9 months in the CXCR7/2+ group, and 6 months in the CXCR7/3+ group, while the median time of CRP-FS was 21 months in the CXCR7/1+ group, 12 months in the CXCR7/2+ group, and 6 months in the CXCR7/3+ group, respectively.

**Conclusion:**

Overexpression of CXCR7 induced by an AR antagonist in CRPC patients displays much better treatment response to Enza. CXCR7 might be a novel therapeutic target gene for CRPC patients.

## 1. Introduction

Substantial researches demonstrate that the androgen receptor (AR) signal pathway is the most important regulatory mechanism governing the malignant progression of prostate cancer (PCa). Activation of AR could enhance the capacity of c-Myc-dependent antiapoptosis and DNA damage repair [1] via several downstream signal pathways, which include Jagged1/Notch1 [2, 3], CXCR4-CXCR7 dimer [4, 5], EGFR/Ras/ERK [6], EGFR/JAK/STAT [7], and EGFR/PI3K/Akt [8]. In addition, some recent reports show that AR could also upregulate DNA damage response and facilitate malignant progression of PCa through the CDC6-ATR-Chk1 pathway [9] and the beta-catenin-ATM-Chk2 pathway [10, 11].

Androgen deprivation therapy (ADT) remains the standard therapy for patients with prostate cancer (PCa). Approximately 80%-90% of men with PCa initially respond to ADT; however, in nearly all cases, the tumors develop resistance to ADT and progress to metastatic castration-resistant prostate cancer (mCRPC) [12]. Enzalutamide (Enza) is a novel 2nd generation androgen receptor (AR) antagonist, which maximally suppresses androgenic signaling and prolongs survival by 32.4 months [13]. However, after being widely used in clinical practice, Enza was shown to be insensitive for about 42% of CRPC patients [14]. Despite initially high response rates, nearly all men eventually developed resistance to Enza after about 11.2 months [13]. Therefore, the challenge of overcoming Enza resistance has emerged as an important topic in the field of CRPC treatment. And it also indicates the need for a deeper understanding of the molecular mechanisms of Enza resistance and the development of more effective predictive biomarkers and therapeutic approaches for CRPC.

Our previous study [15] has confirmed that CXCR7 derepression is closely associated with Enza resistance. When AR activity is blocked by Enza, CXCR7 begins to derepress from AR inhibition, and further drives CRPC cell progression through enhancing antiapoptosis, rapid proliferation, DNA repair, and angiogenesis. After being combined with the CXCR7 antagonist (CCX771), Enza treatment response was significantly improved in CRPC cellular and animal models. In the present study, we attempt to further confirm whether CXCR7 derepression could predict Enza treatment response in CRPC patients and assess whether CXCR7 indicates an AR-independent signal pathway, which might contribute to overcoming Enza resistance and improving the oncological outcome of CRPC patients.

## 2. Patients and Methods

### 2.1. Patients

All eligible cases with CRPC, who were diagnosed and treated in Beijing Anzhen Hospital from January 2015 to December 2019, were recruited into this study. The follow-up period ended in June 2020.

#### 2.1.1. Inclusion Criteria

Inclusion criteria in this trial were based on EAU guidelines. A patient meeting the following factors in these inclusion criteria was diagnosed as CRPC: castrate serum testosterone < 50 ng/dL or 1.7 nmol/L plus either three consecutive rises in PSA at least one week apart resulting in two 50% increases over the nadir and a PSA > 2 ng/mL or the appearance of new lesions, either two or more new bone lesions on bone scan or a soft tissue lesion.

#### 2.1.2. Exclusion Criteria

Exclusion criteria include previous radical prostatectomy (RP), previous docetaxel-based chemotherapy, previous new endocrine therapies (including Enza or abiraterone), ECOG ≥ 3, other malignant tumors, severe cardiopulmonary disease, and interruption of follow-up. This study was approved by the local Ethics Committee of the Capital Medical University. Written informed consent was obtained from each study participant.

### 2.2. Immunohistochemical Staining

Tumor tissue samples were acquired by ultrasound-guided puncture biopsy from CRPC patients. Six specimens were collected from each case, which were then fixed in 4% paraformaldehyde for at least 24 h. Subsequently, specimens were successively dehydrated by ethanol and treated by xylene. Finally, specimens were embedded by paraffin and prepared as 2 *μ*m thick slices for immunohistochemical staining. Furthermore, CXCR7 immunohistochemistry was carried out on formalin-fixed and paraffin-embedded tissue sections. After tissue sections were deparaffinized and rehydrated through graded alcohol, they were heated by microwave in 0.01 mol/L citrate buffer at pH 6.0 for 10 min to retrieve antigens. Following a 30 min incubation in Dako protein blockage solution, tissue sections were incubated in rabbit polyclonal antibody against CXCR7 (1 : 250; Abcam, Cambridge, MA, USA) for 90 min, followed by incubation in a HRP polymer-conjugated secondary antibody (Dako, Glostrup, Denmark) for 40 min. The immunoreaction was visualized in DAB/H_2_O_2_. The specificity of immunoreactions was verified by replacing the primary antibodies with PBS. Ten high-power fields were selected randomly in slides of each sample, and staining score of CXCR7 was calculated using the following method: staining intensity score was assessed as 0 point for cytoplasmic negative staining, 1 point for mild brown staining, 2 points for moderate brown staining, and 3 points for strong brown staining; and staining extent score was assessed as 0 point for positive cell percentage < 5%, 1 point for 5%~25%, 2 points for 26%-50%, and 3 points for >50%. Finally, staining score of CXCR7 was calculated by intensity score times extent score as follows: (-) for 0 points, (1+) for 1~3 points, (2+) for 4~5 points, and (3+) for ≥6 points.

### 2.3. Treatment and Follow-Up Protocol

All enrolled cases received Enza treatment (160 mg, Qd). The patients were monitored by detecting serum PSA levels every month for the first two years, every 3 months for the next three years, and then annually thereafter. In addition, cranial computed tomography (CT) or magnetic resonance imaging (MRI) was performed to access disease progression and recurrence every 3 months for the first 2 years, every 6 months for the next 3 years, and yearly in subsequent years. Then, bone radioisotope scanning was generally performed every 12 months in follow-up years.

### 2.4. Study Endpoints

During follow-up, we measured the maximum changing extent of PSA value compared to baseline. The primary endpoint was PSAP-FS (time to PSA progression, which was identified as PSA increasing by more than 25%, and confirmed again after four weeks). In addition, the secondary endpoint was CRP-FS (time to clinical or radiographic progression, which was indicated by worsened tumor-related symptoms, or more than 20% of increase in the sum of three diameters of space-occupying lesion, or new bone metastases appearance, or occurrence of death).

### 2.5. Statistical Analysis

Prognostic parameters were evaluated by the multivariate Cox regression analysis. PSAP-FS and CRP-FS curves were obtained by the Cox proportional-hazards model and the Kaplan-Meier analysis. Background characteristics were assessed by the chi-square test or the Kruskal-Wallis test, as appropriate. *P* < 0.05 indicated statistical significance. SPSS v20.0 (SPSS, USA) was employed for all statistical analyses.

## 3. Results

### 3.1. Patient Features

**Figure 1:**
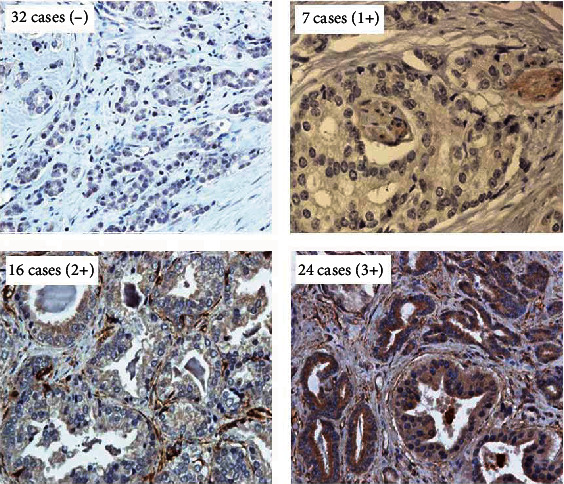
Immunohistochemical staining of CXCR7 in all enrolled CRPC patients. Thirty-two cases negatively expressed the CXCR7 protein, and forty-seven cases exhibited derepressed CXCR7 protein. Among which, seven cases showed weakly positive CXCR7 expression, sixteen cases showed moderately positive expression of CXCR7, and twenty-four cases were strongly positive for CXCR7 expression (magnification: 400x).

A total of 79 patients with CRPC were enrolled in this study. The detailed clinicopathological features are shown in Table 1. As displayed in Figure 1, 32 cases were CXCR7-negative expression as demonstrated by IHC staining in prostatic punctual tissues, and 47 cases were CXCR7-positive expression. Among all CXCR7-positive patients, 7 cases were CXCR7 1+ expression, 16 cases were 2+, and 24 cases were 3+. There was no significant difference between the CXCR7-negative group and the CXCR7-positive group in age at diagnosis, previous treatment, PSA baseline, Gleason score, lymph node invasion, and distant metastasis.

### 3.2. Correlation between CXCR7 Expression and PSA Treatment Response

**Figure 2:**
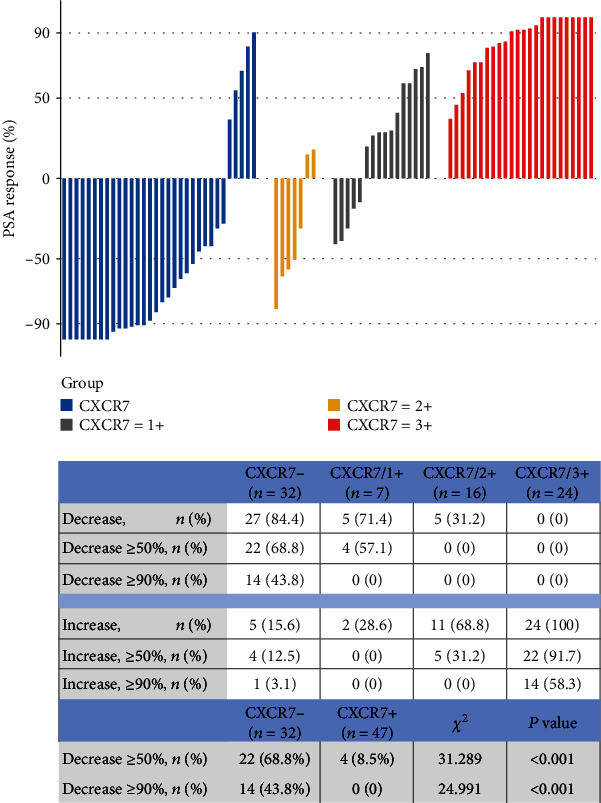
The PSA response to enzalutamide treatment was concluded from all enrolled patients with different expression patterns of CXCR7.

As displayed in Figure 2, the patients with lower CXCR7 expression showed much better PSA response to Enza treatment. The percentage of patients with decreasing PSA was 84.4% in the CXCR7- group, 71.4% in the CXCR7/1+ group, 31.2% in the CXCR7/2+ group, and 0 in the CXCR7/3+ group. Especially, the percentage of patients with increasing PSA was only 15.6% in the CXCR7- group, while the percentage was 68.8% in the CXCR7/2+ group and 100% in the CXCR7/3+ group, respectively. The percentage of patients decreasing over 50% is much higher in the CXCR7-negative group than in the CXCR7-positive group (68.8% vs. 8.5%, *P* < 0.001), and the percentage of cases decreasing over 90% is also significantly higher in the CXCR7-negative group than in the CXCR7-positive group (43.8% vs. 0, *P* < 0.001).

### 3.3. CXCR7-Negative Expression Indicates Better PSA Progression Outcome

**Figure 3:**
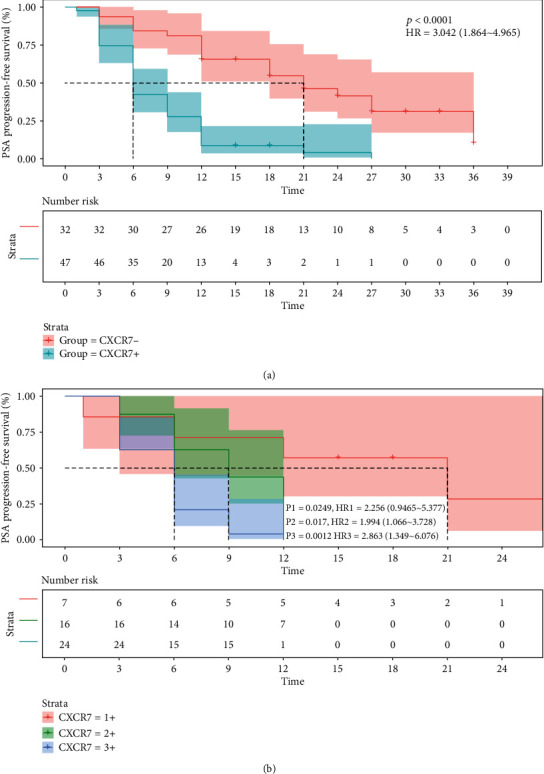
The Kaplan-Meier curves for PSA progression-free survival outcome for all Enza-treated CRPC patients with different expression patterns of CXCR7 (P: CXCR7- vs. CXCR7+; P1: CXCR7/1+ vs. CXCR7/2+; P2: CXCR7/2+ vs. CXCR7/3+; P3: CXCR7/1+ vs. CXCR7/3+).

As shown in Figure 3(a), PSA-free progression rates in the CXCR7-negative group were starkly elevated by Enza treatment compared with those of the CXCR7-positive group at 6 months (84.4% vs. 42.6%), 12 months (65.6% vs. 8.5%), 24 months (31.2% vs. 4.3%), and 36 months (10.4% vs. 0). And median PSA-FS times were 21 months in the CXCR7-negative group and 6 months in the CXCR7-positive group, respectively (*P* < 0.0001, HR = 3.042 (1.864-4.965)). Furthermore, when we stratified the CXCR7-positive group according to positive degree, we found in Figure 3(b) that after Enza treatment, the higher positive degree of CXCR7 expression was significantly associated with the lower PSA-free progression rates. The median PSA-FS time was 21 months in the CXCR7/1+ group, 9 months in the CXCR7/2+ group, and 6 months in the CXCR7/3+ group, respectively (CXCR7/1+ vs. CXCR7/2+: P1 = 0.0249, HR1 = 2.256 (0.9465-5.377); CXCR7/2+ vs. CXCR7/3+: P2 = 0.0017, HR2 = 1.994 (1.066-3.728); CXCR7/1+ vs. CXCR7/3+: P3 = 0.0012, HR3 = 2.863 (1.349-6.076)).

### 3.4. CXCR7-Negative Expression Indicates Better Clinical/Radiographic Progression Outcome

**Figure 4:**
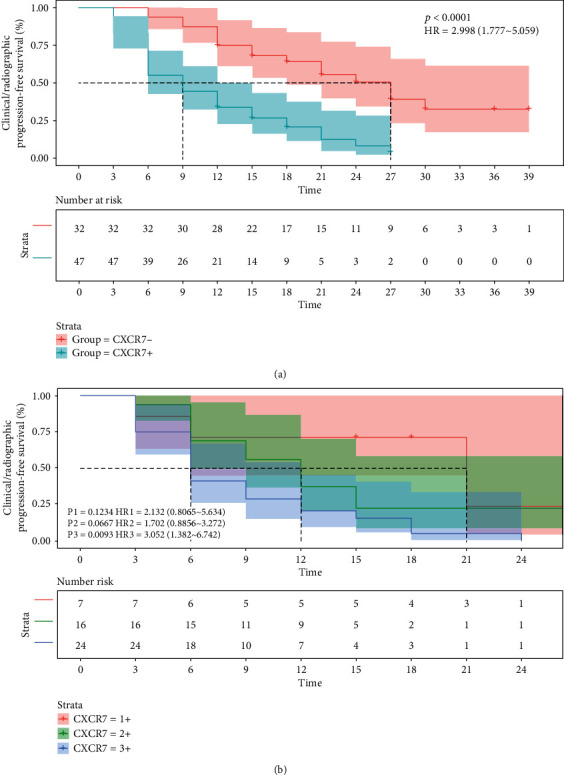
The Kaplan-Meier curves for clinical or radiographic progression-free survival outcomes for all Enza-treated CRPC patients with different expression patterns of CXCR7 (P: CXCR7- vs. CXCR7+; P1: CXCR7/1+ vs. CXCR7/2+; P2: CXCR7/2+ vs. CXCR7/3+; P3: CXCR7/1+ vs. CXCR7/3+).

After Enza treatment, CRP rates between the two different CXCR7 expression groups were compared. As shown in Figure 4(a), remarkably higher values were obtained in the CXCR7-negative group in comparison with the CXCR7-positive group at 6 months (93.8% vs. 55.3%), 12 months (75.0% vs. 34.0%), 24 months (50.6% vs. 8.3%), and 36 months (32.8% vs. 0). The median CRP-FS was starkly increased in the CXCR7-negative group compared with the CXCR7-positive group (27 months vs. 9 months, *P* < 0.0001, HR = 2.998 (1.777-5.059)). As demonstrated in Figure 4(b), stratified analysis further revealed that the patients with higher CXCR7 expression showed much poorer CRP outcome. The median CRP-FS time was 21 months in the CXCR7/1+ group, 12 months in the CXCR7/2+ group, and 6 months in the CXCR7/3+ group, respectively. Although the CRP-FS rate of the CXCR7/2+ group showed no difference with that of the CXCR7/1+ group (P1 = 0.1234, HR1 = 2.132 (0.8065-5.634)) or the CXCR7/3+ group (P2 = 0.0667, HR2 = 1.702 (0.8856-3.272)), there was significant difference of CRP-FS rate between the CXCR7/1+ group and the CXCR7/3+ group (P3 = 0.0012, HR3 = 2.863 (1.349-6.076)).

## 4. Discussion

Enza could maximally inhibit AR signaling and suppress several AR-regulated prooncogenic signaling pathways, yet it also upregulates specific AR-independent signaling pathways that promote malignant progression and rapid development of resistance [16–20]. Enza resistance strongly suggests the existence of alternative signaling pathways beyond that of AR, which triggers unlimited progression of CRPC.

Extensive researches suggest that activation of AR-independent signaling pathways plays critical roles in the progression of metastatic disease and resistance to an AR antagonist. For example, PI3K-AKT-mTOR [21], NF-*κ*B/P52 [22], HER2/HER3 [23], and TGF-*β*1/STAT3 [24] signaling pathways were confirmed to be associated with Enza resistance. Additionally, several reports also found in the AR antagonist-resistant model that elevated expression of glucocorticoid receptors [25], enhanced neuroendocrine transformation [26], and autophagic potency [27], may be governing the development of Enza-resistant CRPC. In 2013, Li et al. confirmed that AR-v7, which is the AR cleavage variant, was responsible for Enza resistance in CRPC cells. By knocking-out AR-v7, cell proliferation and differentiation were both inhibited [28]. Antonarakis et al. further identified positive expression of AR-v7 in circulating tumor cells of clinical CRPC cases who were resistant to Enza [29]. Therefore, the AR variant may well be one important underlying Enza resistance mechanism.

A lot of studies suggest that among many risk factors, inflammation plays an important role in the development and progression of primary PCa to metastatic disease, and chemokines and the chemokine receptor network are fundamental components of the complex interactions between inflammatory cells and PCa cells [16]. CXCL12 is a homeostatic chemokine that is highly expressed and secreted within the tumor-associated hypoxic and proangiogenic environment and during the autoimmune disease-related activities [30]. Meanwhile, CXCR4, the regular CXCL12 receptor, is highly expressed in aggressive tumors and metastatic PCa cells, which has been linked to PCa bone metastasis, and has been shown to be an independent prognostic biomarker for poor survival [31–34]. CXCL12-CXCR4 signaling has been demonstrated to be the direct link to PCa adhesion, migration, metalloproteinase expression, and invasion [35, 36]. Moreover, CXCR12-CXCR4 signaling plays a key role in the maintenance of PCa stem-like cells, and it is activated in PCa cells that are drug-resistant and contribute to tumor relapse [37].

However, CXCR12-CXCR4 signaling can be significantly inhibited by ADT though it is a critical pathway in PCa progression. Therefore, alternative functional signaling pathways in ADT-treated PCa become extremely important to further understand CRPC development. CXCR7, an alternative CXCL12 receptor [38], was recently shown to have a 9- to 10-fold higher affinity for CXCL12 than CXCR4 [39–41]. CXCR7 expression has a positive correlation with Gleason grade in PCa, and it is also high in PCa metastatic lesions, including soft tissues and bone [42]. CXCL12-CXCR7 signaling was shown to contribute to PCa invasiveness through regulation of CD44 and cadherin-11 [42]. Additionally, Balabanian et al. showed that CXCR7 can activate AKT signaling and upregulate secretion of IL-8 and VEGF, potentially contributing to tumor angiogenesis [42]. Further studies showed that IL-8 treatment can upregulate CXCR7, suggesting an IL-8-CXCR7 positive feedback loop [42, 43]. Remarkably, CXCR7 was reported to interact with EGFR and stimulate increased levels of phospho-EGFR and phospho-ERK1/2 [43]. These results are further supported by a recent study that demonstrates CXCR7-mediated EGFR activation in mouse embryo fibroblasts [4]. The complicated interactions between CXCR7 and other cytokines, the potential positive feedback loop of IL-8-CXCR7, and the intersection of CXCR7-EGFR activation in PCa cells, suggest a specific and potentially therapeutically targetable CXCR7-mediated signaling pathway in PCa.

Our previous study [15] comparatively analyzed the DNA profile of C4-2B cells (an androgen-independent PCa cell line) and Enza-treated C4-2B cells. CXCR7 was demonstrated as a significant overexpression gene in Enza-treated cells. Subsequent functional trials further confirmed that the CXCR7 inhibitor could remarkably suppress cellular proliferation, invasion, migration, DNA damage response, and angiogenesis in *in vitro* CRPC and *in vivo* PDX models. When combining treatment of CXCR7 inhibitor and Enza, a synergistic therapeutic effect could be conspicuously observed in CRPC models. Based on these data, we speculated that CXCR7 may represent one important underlying alternative signaling pathway and valuable biomarker for Enza-resistant progression in CRPC. Recent publications confirmed similar conclusions that CXCR7 overexpression could lead to Enza resistance via activation of the MAPK or Akt pathway, and CXCR7-targeting blockade inhibits CRPC tumor growth and potentially prevents metastasis [44, 45].

Seventy-nine CRPC patients without radical prostatectomy were enrolled into the present clinical study, and the expression of CXCR7 was detected in prostatic biopsy tissues. We further analyzed the correlation of CXCR7 expression with Enza treatment response. After 42 months of follow-up, we demonstrate that CXCR7-positive expression indicates significant resistance to Enza treatment and extremely poor outcomes of PSA progression and clinical-radiographic progression. However, this current study had some limitations, including a single-center trial design and a small cohort size. In a future study, we will test the predictive value of CXCR7 expression to Enza treatment response in a much bigger cohort of CRPC patients who underwent ADT or radiotherapy. In addition, detection of CXCR7 from prostatic biopsy samples limited the wide use of CXCR7 in those CRPC cases who underwent RP treatment. Although circulating tumor cells may be used for the detection of CXCR7 derepression in blood samples, we found that the detection rate of circulating tumor cells was too low for further investigation in our preliminary research. Therefore, how to measure CXCR7 derepression in a blood sample is still a severe technical problem, which seriously limited the use of several Enza-resistant biomarkers in those CRPC patients who underwent RP treatment.

## 5. Conclusion

There remain many mechanisms for CRPC endocrine therapy resistance that are worth exploring. CXCR7 is one of the most important and novel targets that are expected to overcome Enza resistance. An in-depth study and a clearer understanding of the CXCR7 signal transduction pathway components will contribute to the development of new therapeutic drugs to improve the resistance status of Enza in patients presenting with CRPC.

## Figures and Tables

**Table 1:** Detailed clinicopathological features of Enza-treated CRPC patients with different CXCR7 expression profile.

Biomarker	CXCR7+ (*n* = 47 )	CXCR7- (*n* = 32)	Statistical analysis	
	Median (range)	Median (range)	*χ* ^2^	*P* value
Age at diagnosis (years)	74 (67~84)	75 (66~81)	0.275	0.9112
Follow-up (months)	24 (12~42)	28.5 (12~42)	0.637	0.3926
	Count (%)	Count (%)	*χ* ^2^	*P* value
Previous treatment				
Brachytherapy	21 (44.7)	12 (37.5)	3.398	0.1829
Radical radiotherapy	9 (19.1)	12 (37.5)		
Endocrine therapy	17 (36.2)	8 (25.0)		
Serum PSA (ng/mL)				
≤20	8 (17.0)	6 (18.8)	0.296	0.8624
20~50	17 (36.2)	13 (40.6)		
≥50	22 (46.8)	13 (40.6)		
Gleason score				
≤6	1 (2.1)	0 (0)	2.463	0.2919
=7	2 (4.3)	4 (12.5)		
≥8	44 (93.6)	28 (87.5)		
Lymph node metastasis				
Yes	13 (27.7)	11 (34.4)	0.406	0.5240
No	34 (72.3)	21 (65.6)		
Distant metastasis				
Yes	7 (14.9)	9 (28.1)	2.064	0.1508
No	40 (85.1)	23 (71.9)		

Note: all cases were strictly accordance with the inclusion and exclusion criteria.

## Data Availability

The analyzed data sets generated during the study are available from the corresponding author on reasonable request.
